# Frontal two-electrode transcranial direct current stimulation protocols may not affect performance on a combined flanker Go/No-Go task

**DOI:** 10.1038/s41598-023-39161-y

**Published:** 2023-07-24

**Authors:** Adrienn Holczer, Teodóra Vékony, Péter Klivényi, Anita Must

**Affiliations:** 1 Department of Neurology, Faculty of Medicine, Albert Szent-Györgyi Health Centre, University of Szeged, Semmelweis u. 6, Szeged, Hungary; 2 Centre de Recherche en Neurosciences de Lyon CRNL U1028 UMR5292, Université Claude Bernard Lyon 1, CNRS, INSERM, 95 Boulevard Pinel, 69500 Bron, France; 3 Chronos Systems on behalf of WCG Clinical Endpoint Solutions, Budapest, Hungary

**Keywords:** Cognitive control, Attention

## Abstract

Transcranial direct current stimulation (tDCS) has been tested to modulate cognitive control or response inhibition using various electrode montages. However, electrode montages and current polarities have not been systematically compared when examining tDCS effects on cognitive control and response inhibition. In this randomized, sham-controlled study, 38 healthy volunteers were randomly grouped into receiving one session of sham, anodal, and cathodal each in an electrode montage that targeted either the dorsolateral prefrontal cortex (DLPFC) or the fronto-medial (FM) region. Participants performed a combined flanker Go/No-Go task during stimulation. No effect of tDCS was found in the DLPFC and FM groups neither using anodal nor cathodal stimulation. No major adverse effects of tDCS were identified using either montage or stimulation type and the two groups did not differ in terms of the reported sensations. The present study suggests that single-session tDCS delivered in two two-electrode montages might not affect cognitive control or response inhibition, despite using widely popular stimulation parameters. This is in line with the heterogeneous findings in the field and calls for further systematic research to exclude less reliable methods from those with more pronounced effects, identify the determinants of responsiveness, and develop optimal ways to utilize this technique.

## Introduction

Transcranial direct current stimulation (tDCS) has been increasingly tested to modulate a wide range of motor and cognitive functions^1^, including interference control and response inhibition, which are among the core aspects of executive functions and are essential for adaptive behavior^2^. However, the mechanisms through which tDCS influences brain activity and behavior are yet to be completely understood and are modulated by several factors, including study design parameters^3^. One key factor influencing tDCS effects is the electrode montage that determines the direction and magnitude of the current passing through the brain^4,5^.

A number of tDCS studies have targeted the dorsolateral prefrontal cortex (DLPFC) to modulate interference control (operationalized as performance on the flanker or Stroop tasks^6–10^, or response inhibition measured using the Go/No-Go or stop-signal tasks^11–13^) based on its reliable activation while performing these tasks^14–16^. Many studies used an asymmetric electrode montage (i.e. one electrode on the left DLPFC and another over the contralateral supraorbital area)^6–8^ that is frequently used when studying the cognitive effects of tDCS in other domains (e.g.^17–20^). Recent studies have found that anodal tDCS over the DLPFC was associated with improved interference control or enhanced response inhibition^6–8,11,21^, in accordance with the assumption that anodal tDCS depolarizes the neuronal membrane and, thus, increases spontaneous brain activity^22^. However, the efficacy of cathodal tDCS has been questioned regarding the modulation of cognition^23^, and more specifically, interference control and response inhibition^6,13^. Additionally, some reports have found no cognition-modulating effects of either anodal or cathodal tDCS when targeting the DLPFC^9,24,25^.

Importantly, the implementation of both interference control and response inhibition results from the dynamic interplay between several cortical areas^26,27^. Apart from the DLPFC, the anterior cingulate cortex (ACC) is considered a hub for monitoring and detecting interference^28–30^ via engaging with the DLPFC which is associated with interference resolution^15,16^. Increased activity in the fronto-medial areas has also been reported with respect to response inhibition on a Go/No-Go task^27^. Accordingly, studies have targeted different brain sites with various electrode montages with the common aim of improving interference control and response inhibition^8,10,12,31^. Nevertheless, the results have been ambiguous: while only a limited impact on response inhibition has been reported, electrophysiological evidence has suggested that tDCS modulates error-related measures and conflict detection^12,31,32^. The effects of fronto-medial tDCS on stimulus-stimulus interference resolution have not been investigated. Still, considering that interference control also involves an evaluative (i.e. conflict monitoring) phase^33^, fronto-medial tDCS may result in increased conflict monitoring and associated behavioral changes.

To date, montages targeting the DLPFC alone and fronto-medial montages have not been directly compared despite both being commonly used in the field^32,34^. For a clinically meaningful effect, systematic comparisons (with stimulation parameters that are not of interest kept constant) and replication studies are of paramount importance in identifying the most effective parameters^35^. Excluding those sets of parameters that yield inconclusive effects may aid the exploration of methods that are more reliable. In addition, it is also recommended to test polarity specificity, that is, to include both anodal and cathodal stimulation in the experimental paradigm^13^. The present randomized, sham-controlled study compared the effects of anodal, cathodal, and sham tDCS on interference control and response inhibition using a combined flanker Go/No-Go task while contrasting the cognition-modulating effects of two prefrontal electrode montages: a conventional DLPFC and a fronto-medial montage. In addition to cognitive changes, we also monitored adverse effects and compared them across the two montages.

## Materials and methods

### Experimental design

A randomized, sham-controlled mixed-design study was conducted on healthy volunteers. The experiment consisted of three sessions of tDCS (anodal, cathodal, and sham) with a counterbalanced stimulation order that was randomized at the beginning of each participant’s first session using computer-generated allocation. With the same method, participants were randomly assigned to one of two remaining experimental groups. They received stimulation either over the left DLPFC (DLPFC Group) or the fronto-medial (FM) areas (FM Group). The target area was kept constant for a given participant. Immediately after starting the stimulation, participants performed a combined flanker Go/No-Go task detailed below. After each session, participants filled out a questionnaire to assess the presence of any adverse effects. The interval between the different sessions was at least 48 h to avoid potential carryover effects. The study was conducted in accordance with the declaration of Helsinki, and the experimental protocol was approved by the Ethics Committee of Albert Szent-Györgyi Clinical Centre, University of Szeged (Ref No.: 174/2018).

### Participants

40 healthy young subjects (M_age_ = 23.28; years; SD_age_ = 3.46 years R_age_ = 18–31 years) were recruited in our study (20 females). Two participants withdrew participation after the first session: one participant dropped out due to a headache after the first (sham) session, and one participant due to logistical issues after receiving anodal stimulation. As these dropouts were deemed random, the data of 38 participants (complete cases) were analyzed (see Table 1). Overall, 38 participants completed all sessions with a mean of 8.3 days apart. The minimum group size was predefined to include at least 15 participants in accordance with previous studies with similar interventions and outcome measures with significant findings^6,11,36,37^. The participants were naïve to the purpose of the study and were debriefed after the last session ended. All participants had normal or corrected-to-normal vision and met the safety restrictions of tDCS (e.g. lack of history of epilepsy, previous head injury, the presence of metallic implants in the cephalic region, or any implanted electronic devices). None of the participants reported a history of any neurological or psychiatric disorders or the use of any drugs affecting the function of the central nervous system. All participants were informed about the potential side effects of the stimulation and signed an informed consent form prior to the experiment.

**Table 1:** Demographic characteristics of the subgroups.

	DLPFC group (n = 19)	FM group (n = 19)	*p*	BF_01_
Age (mean years of age ± SD)	23.63 ± 3.62	24.00 ± 3.50	0.752	3.048
Sex (m/f)	11/8	13/6	0.737	2.173
Handedness (r/l)	15/4	17/2	0.557	3.058

Between-group analyses were carried out using independent *t* tests for continuous variables and Fisher’s exact tests for categorical variables. BF_01_ indicates the Bayes factor in favor of the null hypothesis over the alternative hypothesis.

### Experimental task and procedure

**Figure 1:**
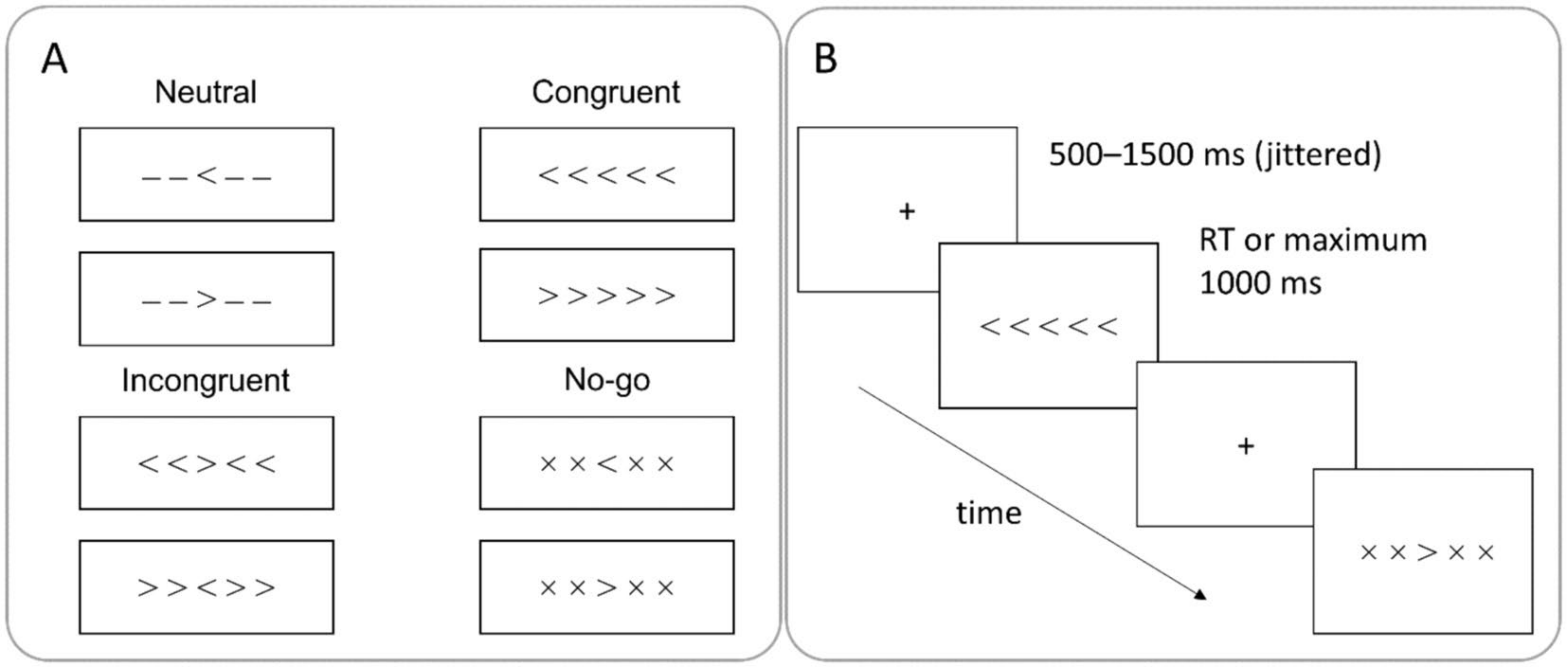
The trial types (**A**) and task flow (**B**) of the combined flanker Go/No-Go task.

A combined version of the Eriksen flanker and the Go/No-Go tasks was used to examine cognitive control performance (Fig. 1), based on the task used by Zmigrod et al.^11^. Combined tasks like ours have been shown to yield comparable behavioral results as well as brain activation patterns as the traditional flanker and Go/No-Go tasks^38–41^. The task was presented using E-Prime version 2.0^42^. An arrow (target stimulus) pointing to the left or the right appeared on the screen, surrounded by four other stimuli. Participants were asked to respond to the middle (target) stimulus with the left or right arrow button of the keyboard with their left or right index finger of each hand, respectively. Based on the characteristics of the surrounding stimuli, four trial types could be differentiated: *congruent* (surrounding stimuli trigger the same response as the target stimulus), *incongruent* (surrounding stimuli trigger a different response as the target stimulus), *neutral* (surrounding stimuli do not indicate orientation), and *no-go* trials (surrounding stimuli indicate response inhibition). In *no-go* trials, participants were instructed to withhold their response when “ × ” symbols surrounded the target stimulus. The stimuli remained on the screen until response or up to 1000 ms. The inter-stimulus interval varied pseudo-randomly between 500 and 1500 ms. The mean inter-trial interval was 625.94 ms (SD = 232.9).

The order of the trials was randomized, and the number of trials for each trial type was counterbalanced. The task was preceded by a practice block containing 16 trials when participants received immediate feedback on their accuracy. After that, six blocks of 96 trials were completed by the participants (576 trials). Between each block, participants could rest and continue the task at their pace.

### tDCS stimulation parameters

**Figure 2:**
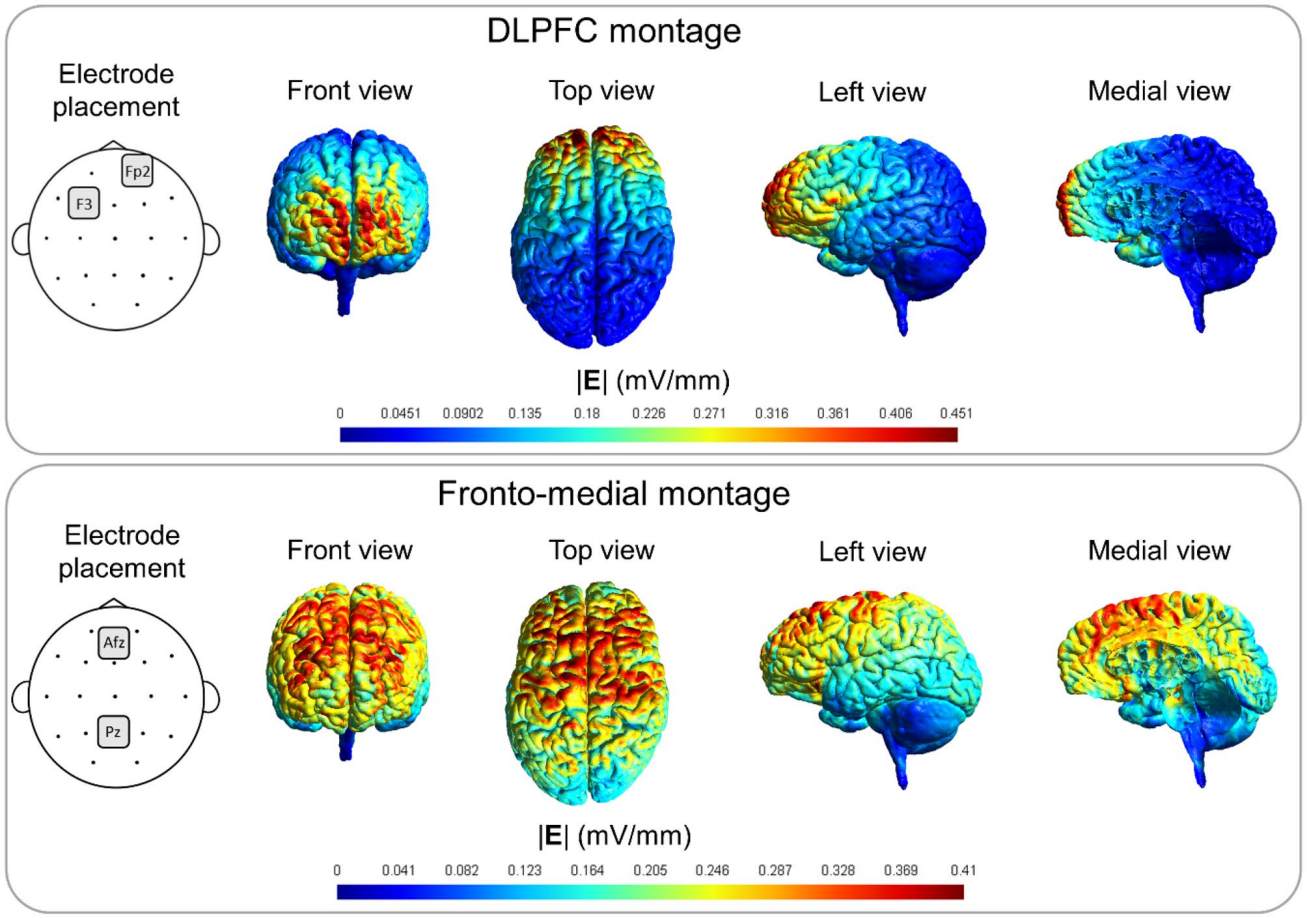
Simulation of normalized electric field distribution (|E|) for both montages. Field strengths were similar between electrode montages. For the anodal fronto-medial stimulation, the anode was applied over the AFz and the cathode over the Pz. For anodal stimulation of the left DLPFC, the anode was placed over the F3 according to the international 10–20 EEG localization system, while the cathode was positioned over the contralateral supraorbital area. When applying cathodal stimulation, the position of the anode and cathode electrodes was reversed.

Stimulation was delivered using the Eldith DC Stimulator Plus (NeuroConn GmbH, Ilmenau, Germany). The stimulation parameters including the electrode size, current intensity, stimulation duration, and sham protocol were chosen based on the most common settings in the literature^34^. Rubber electrodes covered in 35 cm^2^ saline-soaked sponges were fixed on the scalp with plastic traps. Current strength of 2 mA was used. For anodal stimulation of the left DLPFC, the anode was placed over the F3 according to the international 10–20 EEG localization system, while the cathode was positioned over the contralateral supraorbital area. For the anodal fronto-medial stimulation, the anode was applied over the AFz and the cathode over the Pz. Simulation of electric fields generated by tDCS for both electrode montages was also performed to ensure targeting. When applying cathodal stimulation, the position of the anode and cathode electrodes was reversed. To simulate the current flow (Fig. 2), we created three-dimensional head models with a finite element method using SimNIBS v3.2 with the ‘Ernie’ head model^43^. Isotropic conductivities were adopted from the SimNIBS GUI. Twenty minutes of stimulation with 10 s of fade-in and fade-out was carried out for both groups. Sham stimulation was identical to the active protocol of the given group, except that the stimulation length was reduced to 30 s. The position of the anode and cathode during sham stimulation was randomized and counterbalanced across groups.

### Procedure

Participants started the flanker task immediately after the start of the stimulation. The expected length of the cognitive control task was matched with the length of the stimulation. After finishing the task, participants completed a questionnaire regarding the subjective effects and sensations experienced during the stimulation.

### Adverse effects

A self-reported questionnaire recommended by Brunoni et al. was administered to evaluate and compare the adverse effects of both montages^44^. The following symptoms were included in the questionnaire: headache, neck pain, scalp pain, tingling, itching, burning sensation, skin redness, sleepiness, trouble concentrating, and immediate mood changes. Participants were also asked to report if they experienced any symptoms not listed. Symptoms were rated based on presence with a 4-point rating scale (1 = absent, 4 = severe) and certainty (whether the sensation was related to the stimulation or not) with a 5-point rating scale (1 = none, 5 = definite).

### Statistical analysis

Data were analyzed using JASP (version 0.17.2.1.^45^) and the figures were made in R (version 4.0.3^46^). Median reaction times (RTs) of correct trials were entered into a mixed-model analysis of variance (ANOVA). A 2 × 3 × 3 ANOVA was used with Montage (DLPFC, FM) as a between-subject factor and with Stimulation Type (sham, anodal, cathodal) and Trial Type (neutral, congruent, incongruent) as within-subject factors. No-go trials could not be included in the RT analysis as these required the suppression of motor response.

Interference effects were calculated for all stimulation types by extracting the median RTs of the congruent trial type from the median RTs of the incongruent trial type (Interference effect = RT_incongruent_–RT_congruent_). Lower interference effect score indicates better recruitment of cognitive control. Next, a 2 × 3 ANOVA was performed on the interference effect scores with montage (DLPFC, fronto-medial) as a between-subject factor and Stimulation Type (sham, anodal, cathodal) as a within-subject factor. To further explore tDCS effects, congruence sequence effect was analyzed using a 2 × 3 × 2 × 2 ANOVA with Montage (DLPFC, FM) as a between-subject factor and with Stimulation Type (sham, anodal, cathodal), Trial n congruence (congruent, incongruent), and Trial n-1 congruence (congruent, incongruent) as within-subject factors. Due to the low number of trials, CSE was only calculated for the congruent and incongruent trials, and not the Go/No-Go trials. Erroneous trials, the first trials with no previous congruency, and trials preceded by neutral or Go/No-Go trials were removed from the analysis. Thus, four possible categories were possible based on Trial n-1 and Trial n congruence: congruent trials preceded by congruent trials (cC), congruent trials preceded by incongruent trials (iC), incongruent trials preceded by congruent trials (cI) and incongruent trials preceded by incongruent trials (iI).

Accuracy data were analyzed similarly to median RTs, except that accuracy scores of no-go trials were also included in the Trial Type factor (neutral, congruent, incongruent, no-go).

The presence of adverse effects was analyzed in separate mixed analyses of variance with Stimulation type (anodal, cathodal, sham) as within-subject factors and Montage (DLPFC, FM) as a between-subject factor. To assess whether adverse effects were comparable, we evaluated the Stimulation type × Montage interaction for each symptom.

For all ANOVAs, Greenhouse–Geisser correction was used if necessary to correct for non-sphericity, and Bonferroni-corrected post-hoc tests were performed for statistically significant results.

Bayesian statistics with default priors were also performed to supplement the frequentist approach by providing an estimate of evidence strength. Bayesian analyses quantify the relative evidence in favor of the null (H_0_) or alternative hypothesis (H_1_) based on the collected data. We calculated the BF_10_, which is primarily a continuous measure; however, it was interpreted based on the following approximate classification scheme: BF_10_ < 0.1 indicates strong evidence for H_0_, a value between 0.1 and 0.33 indicates substantial evidence for H_0_, while a value between 0.33 and 1 indicates anecdotal evidence for H_0_. Anecdotal evidence supports H_1_ if BF_10_ is between 1 and 3, a value between 3 and 10 indicates substantial evidence for H_1_, and BF_10_ > 10 indicates strong evidence for H_1_^47^. For the Bayesian ANOVAs, the inclusion Bayes Factor (BF_incl_) across matched models is also reported. It quantifies the relative difference between models containing the examined effect and the equivalent models that do not contain it. BF_incl_ is calculated by dividing the sum of the probabilities of the observed data by the sum of the updated probabilities and is interpreted in line with the convention of BF interpretation. The exclusion BF (BF_excl_) can be calculated from the BF_incl_ scores by dividing 1 by the BF_incl_. In order to improve the interpretation of our results, we report both the BF_10_ and BF_01_ scores, as well as the BF_incl_ and the exclusion BF (BF_excl_) scores.

## Results

### Reaction times

**Figure 3:**
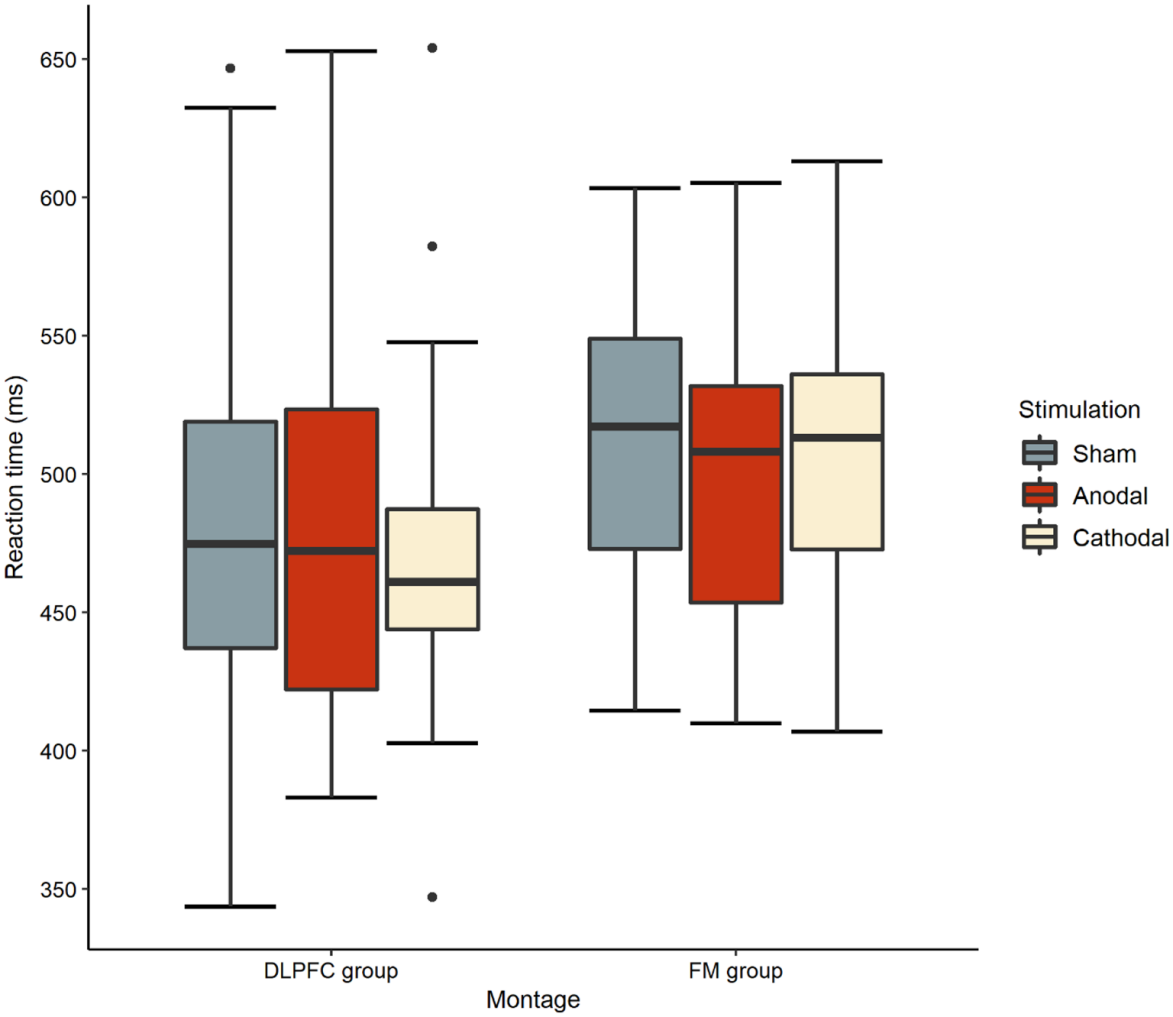
Reaction times per stimulation type in the DLPFC and FM group.

We performed a 2 × 3 × 3 ANOVA with Montage (DLPFC, FM) as a between-subject factor and with Stimulation Type (sham, anodal, cathodal) and Trial Type (neutral, congruent, incongruent) as within-subject factors. The Trial Type main effect was significant, *F*(1.364, 49.111) = 212.611,* p* < 0.001, η_p_^2^ = 0.855, *BF*_incl_ > 10, *BF*_excl_ = 0.1. Post hoc tests showed that RTs were significantly slower in the incongruent trial type as compared to the neutral (*p* < 0.01) and congruent trial types (*p* < 0.01). This result indicates that the flanker task was successful in evoking an interference effect. Bayesian analysis also revealed that Trial Type was the best to predict the data with the highest *BF*_incl_ score suggesting strong evidence to include the effect. The main effect of Stimulation Type and Montage did not reach significance (both *p*s > 0.05; *BF*_incl_ = 0.401 [*BF*_excl_ = 2.493] and 0.395 **[***BF*_excl_ = 2.531**]**, respectively). No interactions were significant by the frequentist analysis methods (all *p*s > 0.05). Bayesian statistics mostly supported these results as the best model only included the main effects of Trial Type and Stimulation type, along with the interaction of Stimulation type and Trial Type. Data were better explained by this model than under the null model (BF_10_ > 10, BF_01_ < 0.1). The *BF*_incl_ score of Trial Type × Stimulation type indicated only anecdotal evidence to include the interaction effect (*BF*_incl_ = 1.926, BF_excl_ = 0.519). Post hoc tests also revealed that RTs did not differ significantly between different stimulation types and montages; although, the median RTs collapsed across the flanker task’s trial types were somewhat elevated in the FM group compared to the DLPFC group (Fig. 3). Please refer to Table 2 for the descriptive data.

**Table 2:** Median RTs in milliseconds and accuracy rates (with standard deviations in parentheses) as a function of electrode montage, tDCS condition and trial type.

	DLPFC montage			Fronto-medial montage		
	Anodal	Cathodal	Sham	Anodal	Cathodal	Sham
Reaction time						
Congruent	468.97 (79.7)	458.84 (69.1)	466.34 (76.5)	482.05 (53.2)	490.23 (51.3)	496.23 (53.7)
Incongruent	509.10 (73.0)	498.42 (64.4)	510.97 (77.4)	522.81 (60.9)	534.63 (55.4)	540.76 (59.3)
Neutral	471.13 (78.4)	464.13 (67.0)	474.92 (79.6)	481.44 (48.2)	498.81 (55.9)	500.26 (51.3)
Congruency sequence effect						
cC	476.987 (82.680)	469.46 (81.8)	473.37 (84.8)	489.46 (55.8)	501.85 (55.7)	506.96 (61.5)
cI	519.48 (76.3)	513.63 (71.5)	524.42 (85.5)	534.77 (71.0)	548.07 (63.1)	563.27 (65.5)
iC	482.25 (89.7)	470.293 (78.3)	480.35 (89.4)	493.37 58.3)	498.72 (54.6)	512.45 (61.6)
iI	513.33 (80.0)	502.15 (68.6)	517.60 (81.5)	521.16 (64.2)	534.97 (53.3)	537.95 (61.9)
Accuracy						
Congruent	0.988 (0.016)	0.988 (0.023)	0.986 (0.026)	0.990 (0.010)	0.988 (0.014)	0.992 (0.008)
Incongruent	0.975 (0.024)	0.975 (0.020)	0.981 (0.015)	0.980 (0.017)	0.981 (0.015)	0.983 (0.015)
Neutral	0.985 (0.025)	0.985 (0.026)	0.989 (0.018)	0.991 (0.014)	0.986 (0.014)	0.992 (0.010)
No-go	0.951 (0.048)	0.962 (0.034)	0.961 (0.045)	0.967 (0.020)	0.970 (0.020)	0.956 (0.031)

*DLPFC* dorsolateral prefrontal cortex, *cC* congruent trials preceded by congruent trials, *iC* congruent trials preceded by incongruent trials, *cI* incongruent trials preceded by congruent trials, *iI* incongruent trials preceded by incongruent trials.

### Interference effect

**Figure 4:**
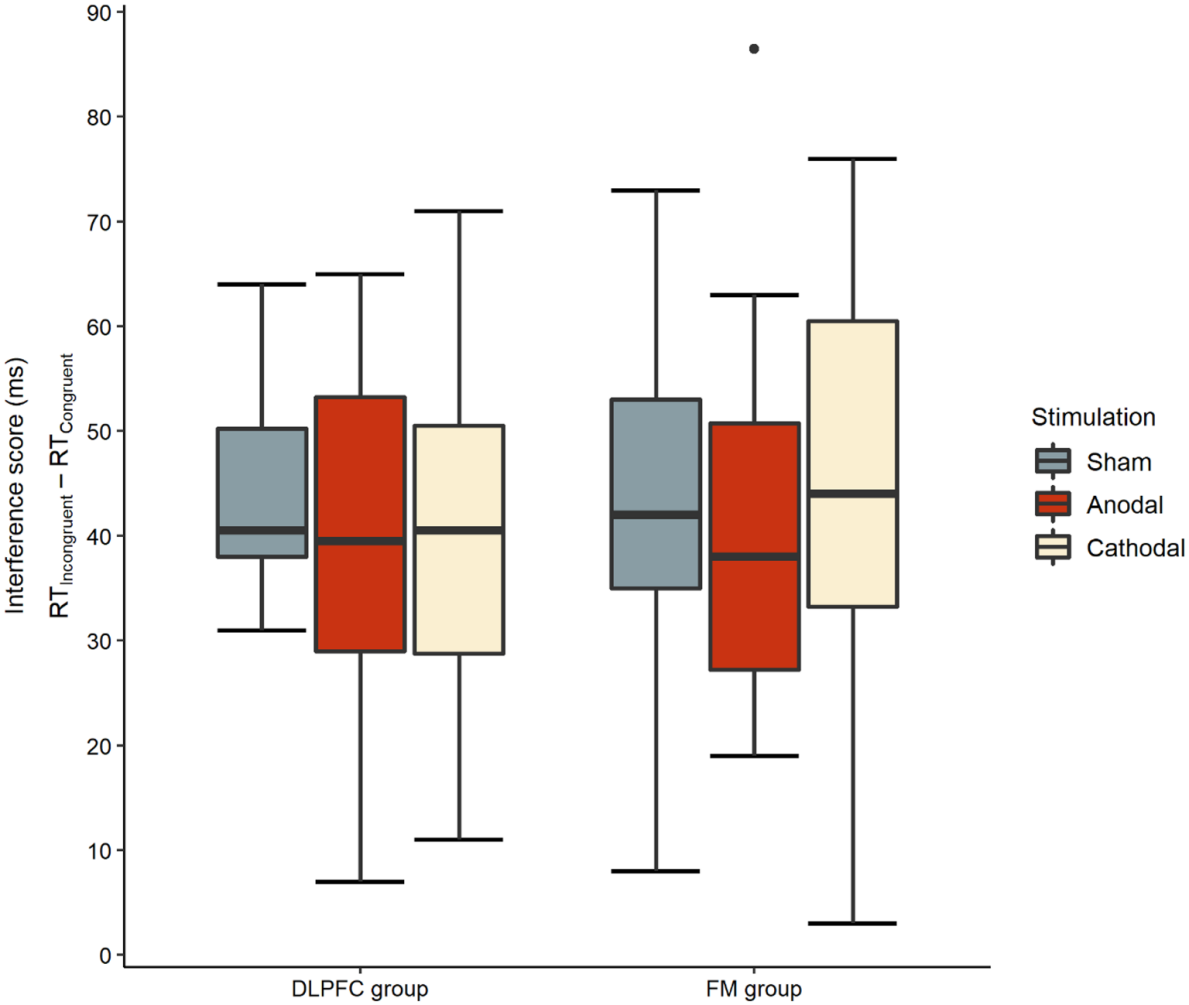
Interference scores per stimulation type in the DLPFC and FM group.

The main effect of Stimulation type, *F*(1.996, 71.846) = 1.882,* p* = 0.160, η_p_^2^ = 0.050, *BF*_incl_ = 0.380, *BF*_excl_ = 2.617, and the main effect of Montage, *F*(1, 36) = 1.704,* p* = 0.704, η_p_^2^ = 0.004, *BF*_incl_ = 0.446, *BF*_excl_ = 2.242, were nonsignificant with *BF*_incl_ scores in favor of H_0_. The interaction of Stimulation type and Montage was also nonsignificant, *F*(1.996, 71.846) = 0.760,* p* = 0.471, η_p_^2^ = 0.021, *BF*_incl_ = 0.246, *BF*_excl_ = 4.065. The Bayesian ANOVA suggested that the null model was the best model also supporting that the included variables did not have a significant effect on the interference scores (Fig. 4).

### Congruency sequence effect

**Figure 5:**
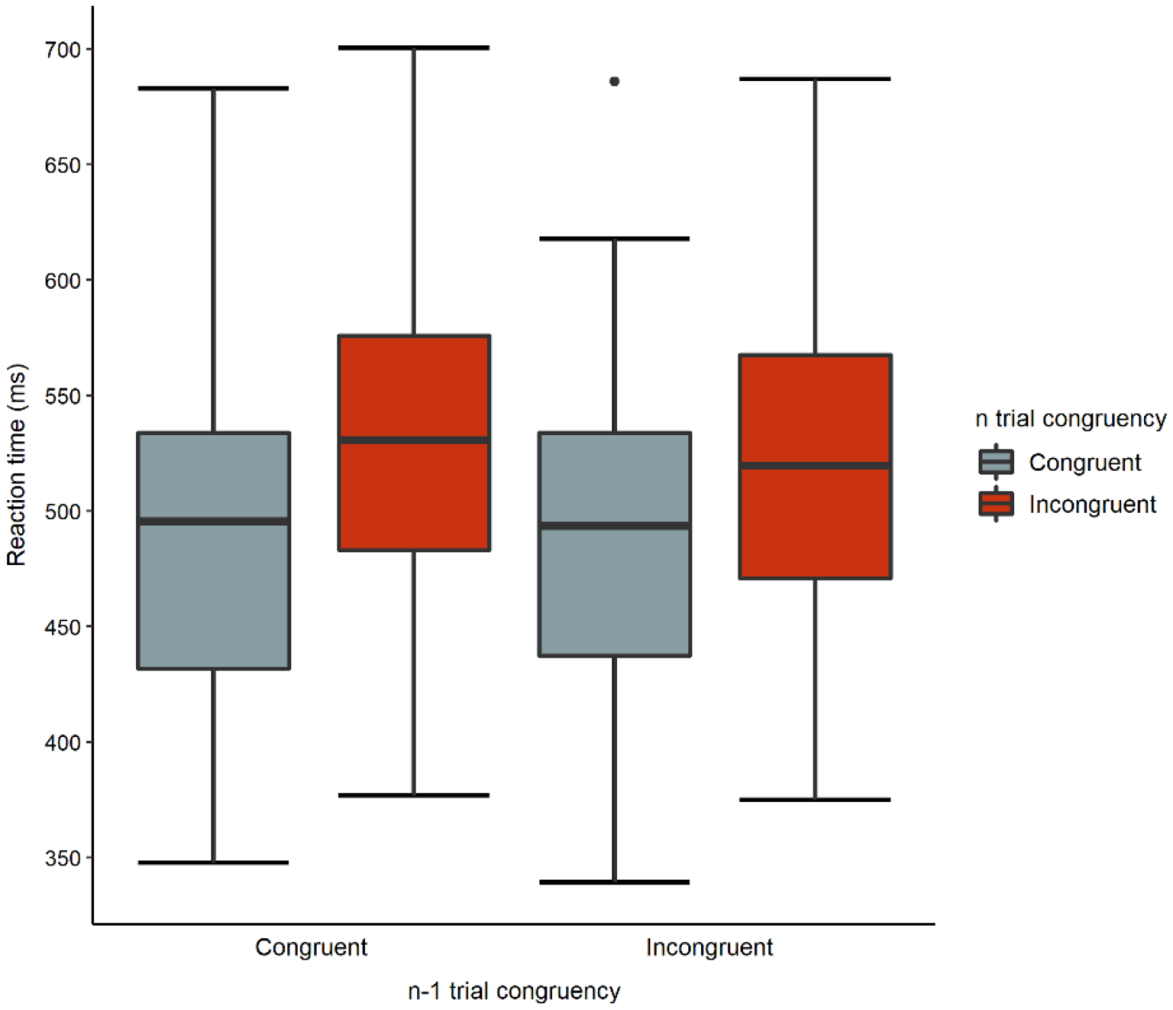
Congruency sequence effects on the combined flanker Go/No-Go task.

The analysis indicated a main effect of Trial n congruency, *F*(1, 36) = 11.573, *p* < 0.002, ηp^2^ = 0.243, *BF*_incl_ > 10, *BF*_excl_ < 10, as well as a main effect of Trial n-1 congruency, *F*(1, 36) = 175.134, *p* = 0.001, ηp^2^ = 0.829, *BF*_incl_ = 9.802, *BF*_excl_ = 0.102, both supported by substantial evidence according to the *BF*_incl/excl_ scores. The former is indicative the presence of the flanker effect in trial n that is also in line with our analysis of the interference effects, while the latter supports that the congruence of trial n-1 has an impact on RTs of trial n. Specifically, RTs were shorter if trial n was congruent as compared to being incongruent (*p* < 0.001), and shorter RTs were recorded when the n-1 trial was incongruent as compared to congruent (*p* = 0.002). The two-way interaction of Trial n-1 congruency × Trial n congruency was also significant (see Fig. 5) which suggests a congruency sequence effect, *F*(1, 36) = 44.125, *p* < 0.0001, ηp^2^ = 0.551, *BF*_incl_ = 83,599.050, *BF*_excl_ = 1.196. Participants were the fastest on cC trials, and these RTs significantly differed from cI and iI trials (*p*s < 0.001), but not from iC (p = 0.509). In turn, RTs on iC were shorter than RTs on either cI or iI (*p*s < 0.001). When incongruent trials were preceded by incongruent trials as compared to congruent trials, RTs were slower (p < 0.001). Another significant two-way interaction was found between Trial n-1 congruency and Montage, *F*(1, 36) = 4.188, *p* = 0.048, ηp^2^ = 0.104, *BF*_incl_ = 0.466, *BF*_excl_ = 2.145. This interaction was primarily linked to the difference of RTs between congruent and incongruent trials on Trial N-1 in the FM group (*p* = 0.003). The rest of the two-way interactions, three-way interactions, and the four-way interaction did not reach significance (all *p*s > 0.005, all *BF*_incl_s < 1.375, *BF*_excl_s < 0.727). The Bayesian analysis supported leaving out these higher-order interactions as the best model only included the main effects, namely, Montage, Stimulation Type, Trial n congruency, and Trial n-1 congruency along with the interaction of Trial n congruence × Trial n-1 interaction. The best model outperformed the null model which supports the inclusion of these factors.

### Accuracy

We detected a near-ceiling effect of performance on the combined flanker Go/No-Go task. The mean overall accuracy was 97.97% (range = 94.73–99.71%). An explanatory ANOVA revealed a significant main effect of Trial Type, *F*(1.166, 41.992) = 14.659, *p* < 0.01, η_p_^2^ = 0.289, *BF*_incl_ > 10, *BF*_excl_ < 0.1, with higher number of errors in the no-go trial type as compared to the neutral, congruent, and incongruent trial type (*p* < 0.05) and no difference between the three latter (*p* > 0.05). No significant interactions were found (*p*s < 0.05, *BF*_incl_s < 0.500, *BF*_excl_ < 2.000).

### Adverse effects

All participants completed the tDCS sessions without major complaints. Participants receiving sham, anodal, and cathodal tDCS in different montages were comparable regarding headache, neck pain, scalp pain, tingling, burning sensation, skin redness, sleepiness, trouble concentrating, and mood changes as the interaction of Montage × Stimulation type was not significant (*p*s > 0.05, *BF*_incl_s < 0.900, *BF*_excl_ > 0.795) (see Supplementary Material). A significant Stimulation type × Montage interaction was found for itching sensation, *F*_2, 72_ = 3.605, *p* = 0.032, η_p_^2^ = 0.091, *BF*_incl_ = 0.729, *BF*_excl_ = 1.371. Post-hoc tests revealed a tendency towards higher levels of itching following anodal stimulation compared to sham stimulation only in the DLPFC group (*p* = 0.011).

## Discussion

Various tDCS electrode montages targeting either the DLPFC or the fronto-medial regions have been utilized to modulate interference control or response inhibition. However, such montages have not been directly compared in terms of efficacy and adverse effects, even though this approach offers insight into their applicability for specific cognitive targets and could aid future study designs. Here, we chose commonly used stimulation parameters^34^ and directly compared a conventional asymmetric DLPFC montage with a fronto-medial montage in a randomized, single-blind, sham-controlled study. We investigated tDCS effects on cognitive control and response inhibition along with adverse effects. Neither anodal nor cathodal stimulation of either montage was found to influence the correct response latency, interference scores, or CSE compared to sham stimulation, when tDCS was delivered for a single session to healthy young adults with the given parameters.

Our findings (supported by both conventional and Bayesian statistical methods) are in line with the incomprehensive results of the literature^3,9,25,48^. We also failed to replicate those results that have indicated that tDCS over the left DLPFC in this specific asymmetrical montage results in performance changes during a cognitive control task^7,8^. This might suggest that conventional tDCS methods that are still widely used in the field^49^ yield inconsistent results in modifying cognitive control, and attention might be steered towards novel electrode montages and optimizing parameter settings.

A possible issue with the two-electrode montages when both electrodes are placed on the head (such as those we used here) is that the partial contribution of the return electrode in reducing cortical excitability over the area below the electrode cannot be completely ruled out. The use of extracephalic montages (i.e. when the return electrode is not placed on the head) might eliminate the effect of the return electrode on cortical modulation^50^. Of note, changing the position of the return electrode to an extracephalic location might affect the current flow and result in the stimulation of areas other than the target region^4^. Moreover, increasing the distance between the electrodes might reduce the neuromodulatory effects of tDCS in some montages^4^. Interestingly, changing the return electrode’s position from the contralateral mastoid to the contralateral supraorbital area (while keeping the anode at the same position) has not been found to affect tDCS effects on cognitive control in a previous study^10^ indicating the possibility that the return electrode’s position might be less pronounced in some cases. Another novel method, the use of high-definition tDCS (HD-tDCS) has yielded promising results regarding its modulatory effects on cognitive control and response inhibition on a behavioral or electrophysiological level^36,51–54^.

The parameter space of other stimulation parameters, including currents strength, electrode size, and stimulation timing should also be revisited and systematically tested due to the lack of consensus regarding their effectiveness^13^, especially since some of them seem not to follow a linear trend in exerting an effect on cognition^55^. While it has been suggested that higher intensity is associated with larger cortical excitability enhancement of the primary motor cortex^56^, in another study, no effect of tDCS has been reported on excitability, not even when the intensity was adjusted to the individual baseline excitability^57^. In the domain of working memory, stimulation intensity had no effect on the enhancement caused by tDCS^58^. Although there are numerous tDCS studies by now, a consistent pattern of an optimal constellation of stimulation parameters is yet to emerge. Of note, the parameters in the present study were set based on previous examples of the literature and are among the most common settings^34^. Moreover, a complete within-subject design with both anodal, cathodal, and sham stimulation was performed on the same subjects in order to reach more reliable conclusions.

The choice of target area has also been a parameter of considerable diversity in the field. Although the asymmetric stimulation of the left DLPFC is fairly popular, and the role of the DLPFC is also supported by evidence from neuroimaging and non-invasive brain stimulation studies^51,59^, growing evidence has been suggesting that the right instead of the left DLPFC might be more involved in response inhibition^13,60^. In addition, the right DLPFC has been also linked to interference resolution at the electrophysiological level which, however, was not expressed on a behavioral level^53^. On the other hand, transcranial magnetic stimulation has been found to enhance performance on a Stroop task only when targeting the right DLPFC, not the left DLPFC^61^. In support of the potential involvement of the bilateral DLPFC, an empirical study involving 120 healthy participants concluded that both left and right DLPFC are involved in interference resolution during a Flanker task as tDCS delivered to both sites has resulted in performance improvement compared to both sham stimulation and active tDCS over a control site^51^. It has been proposed that the left and right DLPFC might play different roles in interference resolution. It has also been suggested that the left DLPFC is involved in anticipatory regulation of control, while the right DLPFC is responsible for adaptive control or interference resolution during response selection. These findings highlight the importance of conducting systematic comparisons between stimulation of the left and right DLPFC^60,61^.

Several other brain areas have been proposed as targets for neuromodulation. A neuroimaging study has indicated that the left anterior insula is a region involved in both response inhibition and cognitive control, making this area another potential target for non-invasive brain stimulation in future studies^62^. Importantly, the dorsal anterior cingulate cortex has also been found active during both interference resolution and response inhibition^15,16,27^. However, it is possible that our attempt to stimulate the fronto-medial areas was not effective in reaching the medial surface of the frontal lobe despite our simulation. Alternatively, the lack of behavioral changes is due to the limited focalization of tDCS effects. The current challenge lies in selecting the right electrode montage to target the fronto-medial cortices (and more broadly, the intended target region) due to the diverse and inconsistent findings in the existing literature.

With the advancement of computational modeling, the simulation of electric fields has become more accessible. Recent evidence contradicts the notion that tDCS has the most pronounced effect directly beneath the electrodes^63^. In our study, we employed a conventional asymmetric montage, which has been recently suggested to generate high electric field magnitudes not only over the DLPFC but also over the frontopolar regions^64^. Stimulation of the orbitofrontal cortex was also implicated in another study^65^. Consequently, the modulation of these areas may have contributed to our null results. The frontopolar cortex has been linked to adaptive resolution of interference on a flanker task^66^, while the orbitofrontal cortex is believed to play a role in response inhibition^67^. The potential excitation of these areas may have also contributed to the positive findings of previous studies utilizing the conventional asymmetric DLPFC montage^6–8^. However, in our study, this did not translate into observable behavioral changes.

It is also worth noting that interference resolution and response inhibition have been operationalized in numerous ways in the literature including the Stroop, flanker, Simon, and anti-saccade, as well as the stop-signal and the Go/No-Go tasks, respectively^68^. Despite the clear presence of interference effect at the behavioral level, only a weak association has been identified between performance on tasks believed to measure interference control^69^ and their correlation with real-life activities and self-reported measures^70^. This suggests that task-specific cognitive processes are likely to play a significant role contrary to a domain-general cognitive control. Additionally, it has been demonstrated that changing the task design such as replacing the stimulus type (e.g. letters, arrows) in the flanker task can lead to differences in reaction time and error rate, despite both exerting the flanker effect. Moreover, some modified tasks might not even be producing a reliable interference effect^71–73^. To enhance the ecological validity of these tasks, several novel tasks have been developed, and despite measurable behavior results, the comparability of these tasks with the classical versions is yet to be established^74–76^. Possibly, these tasks differ to some extent regarding the activation of intra- and interregional networks^68^. For example, less lateralized processing of interference has been suggested on the flanker task than on the Stroop task which might indicate the limited comparability of them^68,77^. Notwithstanding, consistent activation of specific brain regions (including the prefrontal and fronto-medial areas which also served as the target regions in our study) across tasks measuring response inhibition and interference control has been demonstrated^62^. However, future studies should consider that not only the task choice but also the specific details of the task might influence tDCS effects. Besides, non-invasive brain stimulation might act on specific indices of cognitive control (such as the interference score or the CSE) differently within the same task corresponding to the fact that some regions are more involved in interference resolution or adaptive control. Although we did not observe the effect of tDCS on any of these indices, it has been previously reported that on a Stroop task, only transcranial magnetic stimulation differentially affected the CSE and not the interference effect^61^.

In the present study, we chose a combined task in order to measure both interference control and response inhibition. This task has been found comparable to the flanker and Go/No-Go tasks with a moderate correlation of convergence validity^38^. Furthermore, training for four consecutive days using a letter version of a combined flanker Go/No-Go task has resulted in a transfer effect on a traditional Go/No-Go task indicating that at least a partial overlap exists^78^. Combined tasks similar to ours have been also found to elicit electrophysiological and MRI activation patterns that are consistent with previous studies using the flanker and Go/No-Go tasks separately^39–41^. However, one limitation of the present task is that combining the two tasks may impact the way participants interact with the task. In the original flanker task, responses to the flanking stimuli are to be inhibited and the target is supposed to be in the center of attention. Whereas in the combined flanker Go/No-Go task, attention should be allocated to the flanking stimuli as well^79^. Participants also need to keep in mind the differing instruction for no-go trials which may increase the working memory demand of the task as compared to the original flanker and Go/No-Go tasks^38^.

Despite the additional cognitive load, the current task design might not be sensitive enough to detect subtle changes or electrophysiological changes that do not reach the behavioral level. This might be especially true considering accuracy which was consistently high in all participants and trial types in the present study. The ceiling effect might be related to null results in healthy subjects as it might prevent tDCS effects from manifesting. By applying both anodal and cathodal stimulation, the latter classically intended to inhibit the target area temporarily, we aimed to disentangle the effects of current polarity and capture the potential performance deteriorating effects of tDCS as opposed to the improvements that might reach an upper limit. Importantly, our results derived from healthy adults (with performance potentially reaching a ceiling effect) cannot necessarily be expanded to other potential groups of participants. Our null results may be attributed to the possibility of compensatory activations occurring in both the stimulated areas and their contralateral counterparts, or within functional networks. It has been proposed that tDCS is rather a tool to improve deficient cognitive processes, and indeed, encouraging results exist indicating that tDCS can reverse the abnormal activity of several networks in mild cognitive impairment and that this change in brain activation was associated with improved performance^80^.

Finally, another source of inconsistencies of tDCS effects has been attributed not only to methodological differences but also subject variables (such as age, sex, certain cognitive status, and genetics), neurophysiological (e.g. cortical excitability), and other factors (time of day)^81^. In this study, an effort was made to recruit a homogenous sample and groups in terms of sex, age, education, and handedness. Participants were also asked to attempt to schedule all sessions at the same time of day for all three sessions. Our results, coupled with the mixed findings of the literature, might also point towards the relevance of these factors and that tDCS effects are more strongly dependent on brain state and inter-individual responsiveness than on the above-listed parameters; however, more research is warranted to disentangle the complex multifactorial influences of various factors on tDCS effects^82^.

Future lines of research should address these speculations by systematically studying individualized parameters and individual predictors of tDCS efficacy on large samples of participants. Provided that some conventional tDCS methods keep yielding inconclusive results, novel electrode montages might also be considered. Studies with such scope could not only significantly contribute to the understanding of the mechanisms of tDCS and its clinical applicability but also the understanding of the neural background of cognitive control and response inhibition.

## Conclusion

In conclusion, here we began to investigate the role of a less examined stimulation parameter, namely, electrode montage, along with current polarity, on interference resolution and response inhibition in a sample of healthy young adults and found no effects of tDCS. As null results are accumulating in the field, there is still room for further systematic research to identify the determinants of responsiveness and optimal ways to utilize this technique to improve cognition.

## Supplementary Information

Supplementary Table 1.

## Data Availability

The datasets generated and analyzed during the current study are available from the corresponding author on reasonable request.
