# Supplementary material for: Frontal two-electrode transcranial direct current stimulation protocols may not affect performance on a combined flanker Go/No-Go task
Source: Sci Rep. 2023 Jul 24;13:11901. doi: 10.1038/s41598-023-39161-y (PMC10366169; doi:10.1038/s41598-023-39161-y)
Supplement: Supplementary file 1 — Supplementary Table 1. [file 41598_2023_39161_MOESM1_ESM.docx]

Supplementary Materials

Frontal two-electrode transcranial direct current stimulation protocols may not affect performance on a combined flanker Go/No-Go task

Adrienn Holczer, Teodóra Vékony, Péter Klivényi, Anita Must

| Symptom | Sum of Squares | Degrees of freedom | Mean Square | F | p | η_p_² | BF_incl_ |
| --- | --- | --- | --- | --- | --- | --- | --- |
| Headache | 0.070 | 2 | 0.035 | 0.202 | 0.818 | 0.006 | 0.116 |
| Neck pain | 0.053 | 1.481 | 0.036 | 0.274 | 0.694 | 0.008 | 0.176 |
| Scalp pain | 0.053 | 2 | 0.026 | 0.238 | 0.789 | 0.007 | 0.161 |
| Tingling | 0.632 | 2 | 0.316 | 0.852 | 0.431 | 0.023 | 0.420 |
| **Itching** | **2.579** | **2** | **1.289** | **3.605** | **0.032** | **0.091** | **0.729** |
| Burning | 0.754 | 2 | 0.377 | 1.307 | 0.277 | 0.035 | 0.264 |
| Redness | 1.316 | 2 | 0.658 | 1.527 | 0.224 | 0.041 | 0.151 |
| Concentration | 0.368 | 2 | 0.184 | 0.617 | 0.543 | 0.017 | 0.226 |
| Mood | 0.281 | 2 | 0.140 | 2.304 | 0.107 | 0.060 | 0.900 |

**Table 1.** Analyses of variance of Stimulus × Montage interactions for each symptom
